# The structure of the folded domain from the signature multifunctional protein ICP27 from herpes simplex virus-1 reveals an intertwined dimer

**DOI:** 10.1038/srep11234

**Published:** 2015-06-11

**Authors:** Richard B. Tunnicliffe, Mitchell Schacht, Colin Levy, Thomas A. Jowitt, Rozanne M. Sandri-Goldin, Alexander P. Golovanov

**Affiliations:** 1 Manchester Institute of Biotechnology, The University of Manchester, Manchester, UK; 2 Faculty of Life Sciences, The University of Manchester, Manchester, UK; 3 Department of Microbiology and Molecular Genetics, School of Medicine, University of California, Irvine, USA

## Abstract

Herpesviruses cause life-long infections by evading the host immune system and establishing latent infections. All mammalian herpesviruses express an essential multifunctional protein that is typified by ICP27 encoded by Herpes Simplex Virus 1. The only region that is conserved among the diverse members of the ICP27 family is a predicted globular domain that has been termed the ICP27 homology domain. Here we present the first crystal structure of the ICP27 homology domain, solved to 1.9 Å resolution. The protein is a homo-dimer, adopting a novel intertwined fold with one CHCC zinc-binding site per monomer. The dimerization, which was independently confirmed by SEC-MALS and AUC, is stabilized by an extensive network of intermolecular contacts, and a domain-swap involving the two N-terminal helices and C-terminal tails. Each monomer contains a lid motif that can clamp the C-terminal tail of its dimeric binding partner against its globular core, without forming any distinct secondary structure elements. The binding interface was probed with point mutations, none of which had a noticeable effect on dimer formation; however deletion of the C-terminal tail region prevented dimer formation *in vivo*. The structure provides a template for future biochemical studies and modelling of ICP27 homologs from other herpesviruses.

Herpesviridae are double-stranded DNA viruses that cause a variety of diseases in humans and animals. All herpesvirus express an essential multifunctional protein from the ICP27 family^1^. Herpes simplex virus 1 (HSV-1) ICP27 is the signature prototype of the family, and it is composed of 512 amino acids. Structure predictions suggest it can be divided into two general regions, a disordered N-terminal region and a globular C-terminal half containing KH domains and zinc fingers^2^,^3^,^4^,^5^. Experimental studies have indeed shown that the N-terminal region, comprising residues 1-160, is unstructured^6^, and contains a short binding epitope of residues 103-110 for interaction with a cellular mRNA export adaptor protein ALYREF^7^, as well as other multiple functional regions (nuclear export sequence, nuclear localization sequence, and RGG box). The C-terminal globular domain found in ICP27 has homologs in all herpesviruses, hence has been termed the ICP27 Homology Domain (IHD), whereas the primary sequences in the unstructured regions are poorly conserved.

One major function of ICP27 is modulating the expression of viral genes at a post-transcriptional level^8^. The protein functions by interacting with RNA via the RGG box in the N-terminal region^9^,^10^ and also binding to cellular proteins to promote the export of intronless viral mRNA via the TAP/NXF1 pathway^11^,^12^. ICP27 also has an inhibitory effect on host splicing, which in turn represses the export of cellular transcripts which largely contain introns^13^. ICP27 has also been reported to trans-activate gene expression in a subset of specific gamma-2 genes such as gC^14^. Additionally, ICP27 can interact with cellular translation initiation factors to promote the translation of some viral transcripts^15^.

ICP27 is predominantly nuclear at early times after infection and it has been found to be associated with splicing complex proteins in its role as an inhibitor of host cell splicing^13^. It also interacts with the C-terminal domain of cellular RNA polymerase II at early times and is involved in the recruitment of RNA polymerase II to HSV-1 replication sites^16^. Beginning at about 5–6 hours after infection ICP27 actively shuttles between the nucleus and cytoplasm in its role in viral mRNA export^17^. ICP27 interacts with the mRNA export adaptor protein ALYREF^7^,^11^ and it also directly interacts with the mRNA exporter receptor TAP/NXF1^18^,^19^. ICP27 has also been found to interact with cellular translation initiation factors^15^ and with the viral DNA replication protein ICP8^20^. A number of ICP27 deletion mutants have been used to map the regions of interaction between ICP27 and its interacting partners. While some of these interactions have been mapped to the N-terminal half of the protein, such as the interaction with ALYREF, SRPK1 and RNA, a number of interactions have been mapped to the C-terminal globular region, including interactions with SR splicing proteins, translation factors, RNA polymerase II and TAP/NXF1, therefore this domain is of crucial functional importance. However, structural data for the folded domain of ICP27 or any homologs has so far been lacking and it is not known how these deletion mutants affect ICP27 structure, making interpretation of their functional effects difficult. Here we present the first crystal structure of the folded domain of ICP27 comprising C-terminal amino acids 241–512. The data establishes the archetype for the novel IHD fold and should inform future functional studies of homologous herpesvirus proteins by allowing more targeted mutations.

## Results

### ICP27 C-terminal domain structure determination

**Figure 1:**
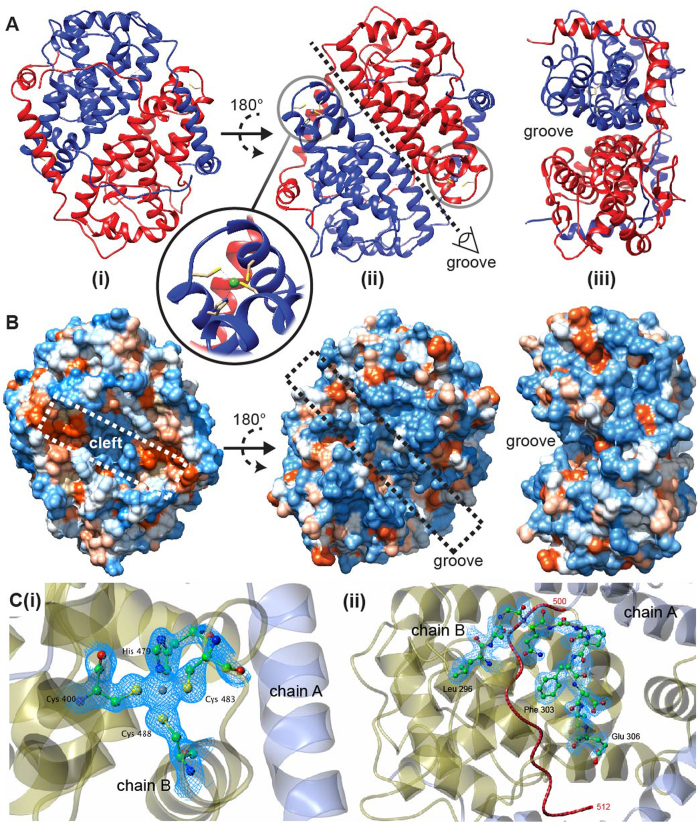
Overview of ICP27Δ241 structure. (**A**) Cartoon representation of the ICP27 domain-swap homo-dimer, protein chains A and B are colored blue and red respectively, with location of a significant groove along the major dimer interface indicated. Grey circle indicates position of CHCC zinc coordination site, with insert showing detail with zinc colored green. (**B**) Surface representation, colored for electrostatic charge: acidic amino acids are colored red, basic blue, and non-charged grey, created with UCSF Chimera^59^ using default Kyte-Doolittle scale^60^. Three molecular orientations are shown, as well as locations of cleft and groove regions. (**C**) Electron density and ball and stick representation of (i) Zn^2+^-binding side chains and (ii) lid region in chain B. The C-terminal residues 500–512 of chain A are colored red. The Zn^2+^-site image was created in CCP4MG^22^, map is 2F_o_-F_c_ contoured at 1.5 sigma level. The lid-site image utilized a Feature Enhanced Map contoured at 1 sigma level, generated in Phenix^23^.

The construct boundaries were chosen based on consensus prediction of the positions of secondary structure elements and disordered regions, to minimize the latter. Three ICP27 constructs, composed of the C-terminal residues 189–512, 215–512 and 241–512 could be purified to homogeneity, however only the shortest construct produced diffracting crystals. ICP27Δ241 readily crystallized in a variety of conditions across 5 commercial screens (JCSG^+^, PACT Premier, Clear Strategy I + II & Morpheus). Initial phases were obtained from single wavelength anomalous dispersion (SAD) experiments carried out on selenomethionine derivatized crystals in Phenix.AutoSol^21^. Subsequent model building and refinement were carried out against a native data set extending to a resolution of 1.92 Å (Table 1). Complete details of the structure determination and model building can be found in the Materials and methods section below. The structure (Fig. 1) revealed two molecules of ICP27 in the asymmetric unit with clearly interpretable electron density for all residues 242–512 of chain B, whilst chain A showed an interpretable trace for residues 242–299 and 303–512 (see representative maps^22^,^23^ on Fig. 1C).

### Structural features of ICP27

**Figure 2:**
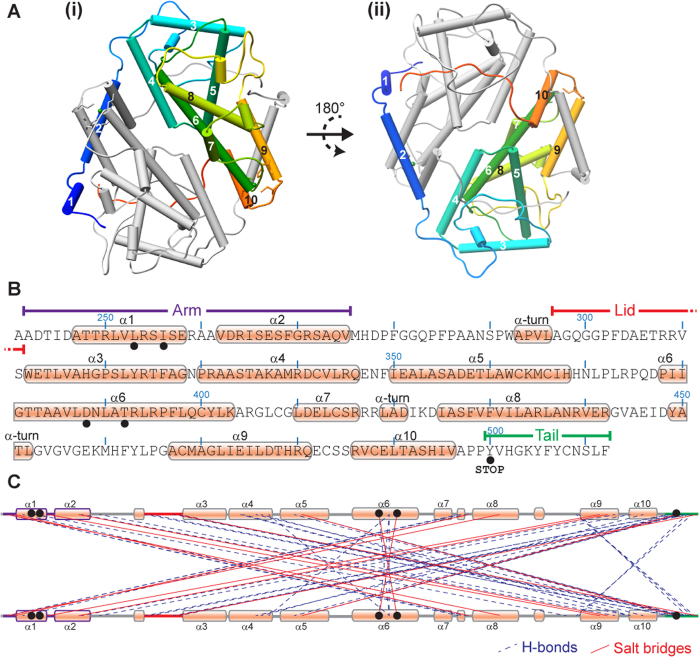
Secondary structure elements and dimerization features in ICP27. (**A**) Location of secondary structure elements in ICP27Δ241, α-helices are shown as cylinders and numbered sequentially; very short helices are not numbered. One chain of the dimer is colored grey while the other colored blue for N-termini through green to red for C-termini. (**B**) Positon of structural features important for dimerization and numbering of α-helices on amino acid sequence. Black dots indicate positions of mutants created for *in vivo* studies (Fig. 6). (**C**) Location of polar intermolecular connectivities holding the dimer together: lines between residues of protein chains indicate the position of salt bridges (red solid lines) and hydrogen bonds (blue dashes). The connectivities were obtained using PDBePISA^24^ web server (http://www.ebi.ac.uk/pdbe/pisa/). Location of amino acid residues probed functionally by point mutations is shown with black dots. The introduction of STOP codon at position 500 led to construct with truncated Tail region.

**Figure 3:**
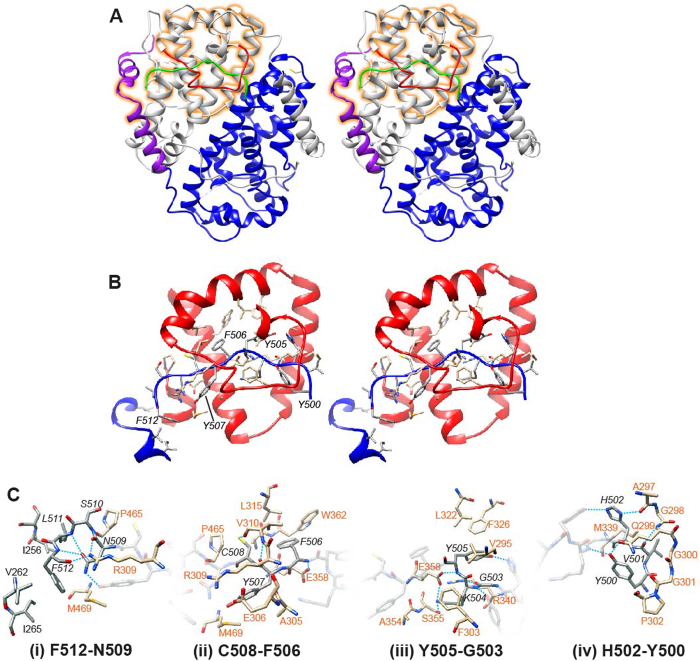
Details of intermolecular contacts with the C-terminal tail region. (**A**) Wall-eyed stereo cartoon representation of ICP27Δ241 structure colored to emphasize the main inter-domain contact motifs. Chain 1 is colored grey, with its Lid motif highlighted in red. Chain 1 interacts with chain 2 colored blue with its Arm colored purple and Tail green. Region highlighted in orange is expanded in panel B. (**B**) Overview of lid-tail interaction, wall-eyed stereo cartoon representation with selected side chains shown as sticks, intermolecular hydrogen bonds and salt bridges are indicated by thin blue lines. Aromatic residues within the tail are labeled. (**C**) Detail of lid-tail interaction for groups of C-terminal tail residues, stick representation with carbon atoms colored grey or orange according to their protein chain, intermolecular hydrogen bonds and salt bridges are indicated by blue dashed lines. Groupings shown are (i) N509-F512, (ii) F506-C508, (iii) G503-Y505 and (iv) Y500-H502, tail residues outside of these respective regions are represented with semi-transparent sticks.

The ICP27 C-terminal domain structure is a homo-dimer, each chain is composed of 10 α-helices, 3 helical turns plus loops. Each chain largely comprises a globular core domain, two of which are closely associated and intertwined forming a compact species with dimensions approximately 65 × 60 × 40 Å (Fig. 1). Numerous intermolecular contacts are made between the two protein chains (Fig. 2), PDBePISA analysis^24^ indicated that 47% of residues observed in the structure have atoms within the dimer interface, and 88 polar intermolecular contacts (62 hydrogen bonds and 26 salt bridges) were detected (Fig. 2B, and Supplementary Information). Only one residue forms an intermolecular contact with its dimeric counterpart, namely T392 situated on helix α6, which forms an inter-chain hydrogen bond via the side chain hydroxyl. The most striking feature of the dimerization is a domain swap comprising two segments that are separated off the core globular domain and interact with the dimeric partner. Firstly, the N-terminal region containing helices α1 and α2 (residues 242–275), adopts an arm-like conformation and embraces the adjacent globular domain (Fig. 2). This is stabilized by four salt bridges formed between the side chains of R259 with E474 and four also between R271 with D477, plus other hydrophobic and ionic contacts. Secondly, the C-terminal tail of 13 residues (500–512) extends into the dimeric partner and becomes clamped under a lid composed of residues 297–311 from the adjacent chain, and also contacts residues in helices α3, α4 and α5 (Fig. 3). A structure comparison search using DALI^25^ indicated a distant structural homology of fragments of a helical bundle part of ICP27 monomer with other helical bundle proteins such as LRR-repeat protein 5 (PDB code 3V5Z), PSB27 (PDB code 2Y6X) and many others; this apparent similarity however was confined to few helices only. No structural homologues for the complete characteristic intertwined dimer fold of ICP27Δ241 were found.

The importance of the C-terminal tail motif for dimer formation (sequence YVHGKYFYCNSLF) is highlighted by the abundance of intermolecular contacts it makes, both with the core and with the lid (Figs 2 and 3). The K504 side chain amine forms a hydrogen bond with S355 side chain hydroxyl and also a buried salt bridge with E358 side chain (Fig. 3C). Also the aliphatic part of the K504 side chain is located under the aromatic ring of F303 (from the lid) forming hydrophobic contacts. Inter-chain hydrophobic interactions occur with the side chains of the aromatic cluster Y505, F506 and Y507. Similarly L511 and F512 side chains make several hydrophobic contacts including for the latter I265 from the arm region. Several intermolecular hydrogen bonds with the tail backbone atoms are also present (Fig. 3, and Supplementary Information).

### Surface features

The surface of ICP27 contains potential binding pockets, which may facilitate interaction with other biological molecules, a groove ~10 Å wide and ~16 Å deep runs along the major dimer interface (Fig. 1). On the same face as the groove, positioned either side, is an extensive cluster of six arginine side chains per monomer - the triplet R416, R417, R418 plus R435, R439 and R442. The function of the basic patch is unknown, it has potential to bind to negatively charged species such as nucleic acids, but a lack of nearby surface exposed aromatic groups makes it difficult to imagine a highly specific interaction. The non-grooved main face of ICP27 contains a relatively shallow cleft (Fig. 1) with two hydrophobic patches, comprising side chains of I265, F269, I350, I473, F512 surface exposed at either end of the cleft suggestive of a binding site.

### Biophysical analysis of ICP27 dimer

**Figure 4:**
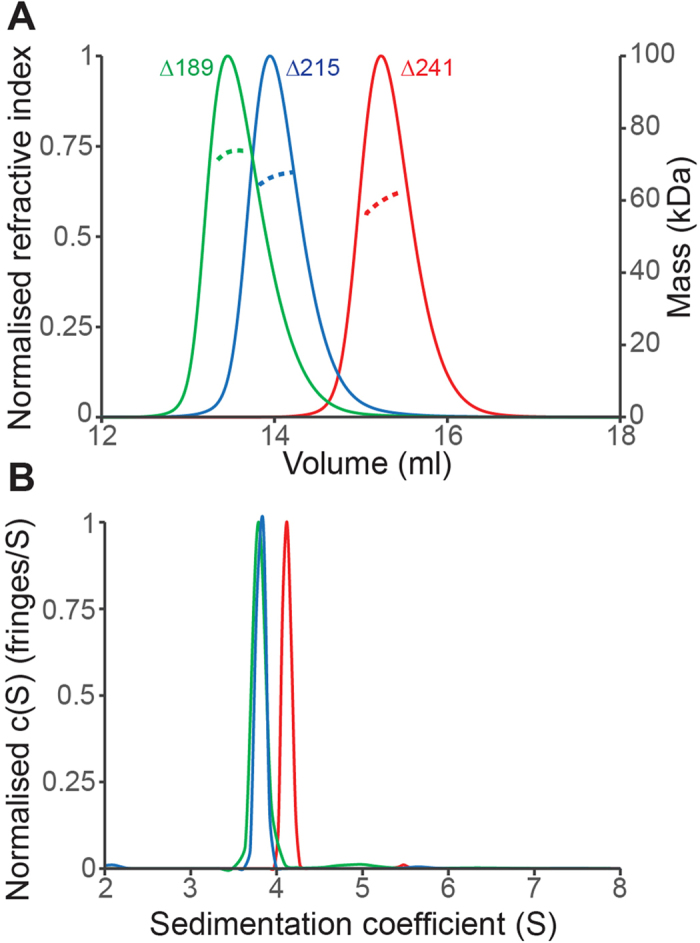
Biophysical evidence of ICP27 homo-dimerization in solution for different constructs. (**A**) SEC-MALS profile of the ICP27Δ241 (red) showing refractive index and molecular mass against elution volume, predicting MW of 60.4 kDa. For comparison, traces for two longer constructs ICP27Δ189 (green) and ICP27Δ215 (blue) are shown which also form dimers in solution. (**B**) Velocity AUC analysis of the ICP27 constructs Δ241, Δ215 and Δ189, colored as in panel A; for each a major peak was observed corresponding to a dimeric species.

In order to confirm that the homo-dimer observed in the crystal structure is present in solution, ICP27Δ241 was analyzed by size exclusion chromatography coupled with multi-angle light scattering (SEC-MALS). The data indicted a species of 60.4 kDa, with hydrodynamic radius of 3.18 nm (Fig. 4A), consistent with homo-dimerization of the predicted 30.1 kDa polypeptide chain of the monomer. Sedimentation analytical ultracentrifugation (AUC) further confirmed dimerization, with calculated molecular weight of 58.6 kDa, the sedimentation coefficient (*S*_20,W_) was measured as 4.23 and frictional ratio (*f/f*_*0*_) = 1.29 (Fig. 4B). MALS and AUC data for longer ICP27 constructs similarly indicated homo-dimerization (Table 2). Therefore the biophysical data consistently show that the constructs encompassing the folded domain of ICP27 used in this study all exist as dimers in solution.

### Zinc coordination

**Figure 5:**
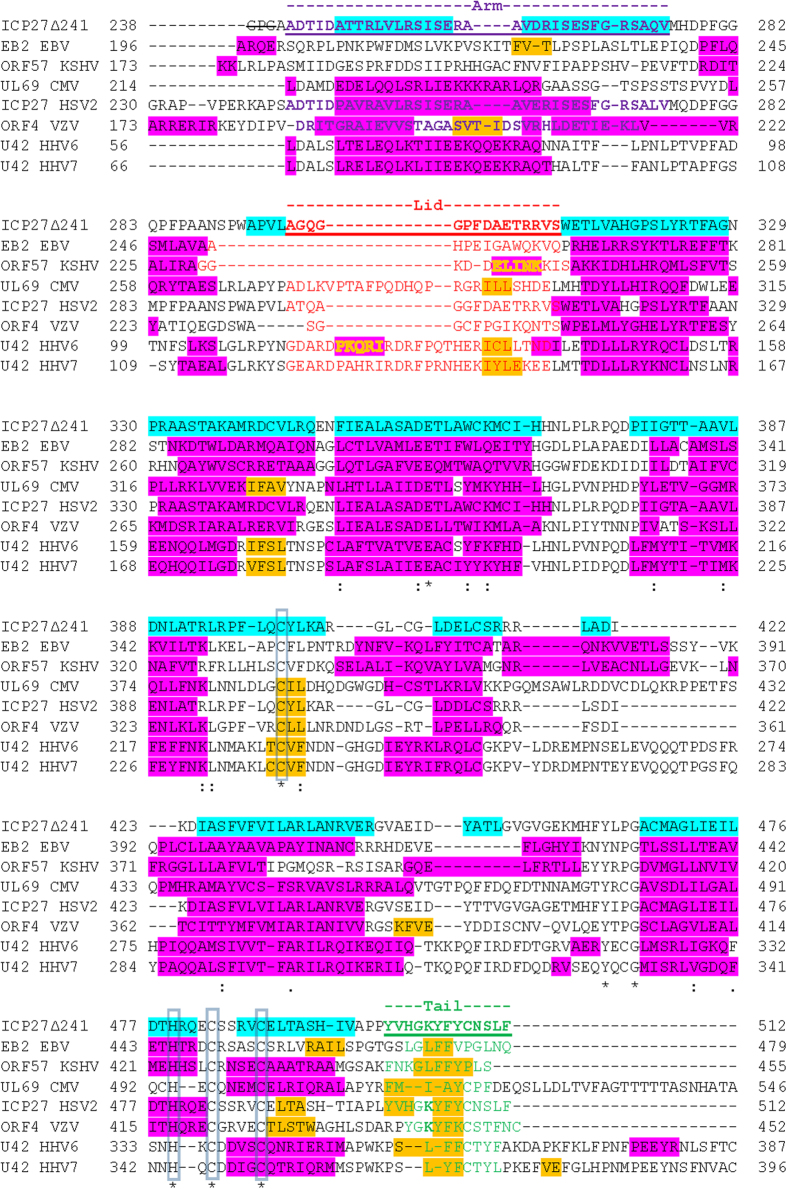
Sequence conservation in ICP27 homologs. Sequences from human herpesvirus homologs of ICP27 aligned with secondary structure elements observed in the crystal structure for HSV1 ICP27Δ241 (shown in turquoise), plus predicted secondary structure elements in other sequences. Predicted α-helices are colored pink and β-sheets orange. Alignment constructed using Clustal omega^28^,^29^ and secondary structure predictions by Psipred^27^ using Uniprot entries P10238 for ICP27 HHV11/HSV1, Q04360 EB2 from EBV, Q2HR75 for ORF58 KSHV, P16749 UL69 CMV, P28276 for ICP27 HHV2, P09269 for ORF4 VZV, P52354 for U42 HHV6, and P52355 for U42 HHV7. The first three residues (GPG) of ICP27Δ241 are not wild type and were introduced during cloning. Positions of residues involved in coordinating zinc are strictly conserved and marked with asterisk and boxes.

Previously zinc binding was observed in ICP27, and the residues C483, C488, H502 and C508 were predicted to form the coordination site^2^,^26^. The structure determined here however revealed a different binding site: each ICP27 chain coordinates a single metal cation in a tetrahedral geometry via the side chains of C400, H479 (via Nε^2^), C483 and C488 (Fig. 1C). These residues, which constitute a CHCC zinc binding site are conserved in all ICP27 homologs (see Fig. 5). Zn^2+^ - S bond lengths in cysteines ranged between 2.23 and 2.40 Å, whereas the Zn^2+^ – Nε^2^ were 2.05 and 2.07 Å. The metal is not surface exposed suggesting its role is primarily structural.

### Features of ICP27 structure conserved in herpesvirus family

To observe the location of conserved features, secondary structure predictions using Psipred^27^ were combined with sequence alignments using Clustal Omega^28^,^29^ of the 8 ICP27 homologs in human herpes viruses (Fig. 5). Interestingly, the zinc-binding CHCC motif is strictly conserved. The patterns responsible for domain-swap features observed for ICP27Δ241 appear in all sequences; the arm and lid are present but only in terms of predicted pair of α-helices and an extended loop respectively, whereas the clusters of hydrophobic and aromatic resides present in the tail are present in all species. The tail includes the GLFF motif previously identified in ICP27 homologs from gamma herpesviridiae (EBV and KSHV), a feature also conserved in beta viruses HHV6 and 7 but not easily identifiable in CMV (Fig. 5). A similar motif is present in alpha sequences (HSV1, HSV2 and VZV) as GKYF, residues 503–506 in ICP27. Additionally an acidic pair (D357+E358 in ICP27) in helix α5 are conserved, these form intra-and inter-molecular salt bridges, for the latter with the tail region (Fig. 3C). At the dimer interface elsewhere only R393 is conserved which in ICP27 forms water mediated hydrogen bonds between the protein chains. The remaining conservation appears in structurally important residues primarily forming the hydrophobic core.

### ICP27 forms dimers during infection

**Figure 6:**
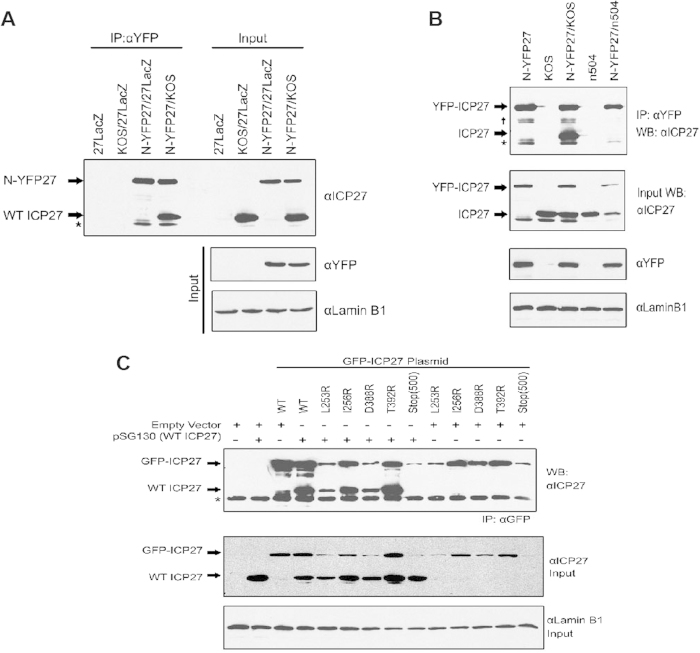
ICP27 forms homo-dimers *in vivo*. (**A**) N-terminally YFP-tagged HSV-1 ICP27 (N-YFP-ICP27^31^) was immunoprecipitated from nuclear extracts of HeLa cells that were infected for 8 hours with 27-LacZ, WT HSV-1 KOS, or N-YFP-ICP27 as indicated using GFP/YFP antibody (Ab290). Western blotting was performed with a combination of ICP27 monoclonal antibodies P1113 and P1119. Blots showing input proteins were also probed with anti-GFP/YFP antibody and the nuclear protein Lamin B1 (EPR8985) as a loading control. Asterisks indicate heavy chain IgG from the immunoprecipitation. (**B**) Nuclear extracts from HeLa cells infected for 8 h with N-YFP-ICP27, KOS, or n504 were immunoprecipitated with anti-GFP/YFP antibody (Ab290). Western blots of the immunoprecipitated samples and input samples were probed with ICP27 monoclonal antibodies (P1113 and P1119). Blots showing input samples were probed with anti-GFP/YFP (Ab290) and Lamin B1. Asterisk marks heavy chain IgG. Dagger marks a non-specific band of unknown origin. (**C**) HeLa cells were co-transfected with pSG130 encoding WT ICP27, empty vector (pUC18), or pGFP-ICP27 plasmids with the indicated C-terminal mutations. ICP27 residues at the indicated positions were substituted with arginine. GFP-tagged ICP27 plasmid Stop500 has a stop codon in all three reading frames at amino acid 500. At 16 hours post transfection, cells were infected with 27-LacZ for 8 hours as previously described^58^. Cell lysates were immunoprecipitated with anti-GFP antibody (Ab1218). Western blots were probed with anti-ICP27 antibodies P113 and P119 and anti-Lamin B1 as indicated. Asterisks indicate heavy chain IgG from the immunoprecipitation.

We showed previously that ICP27 can form dimers or multimers *in vivo* using a yeast two-hybrid assay and co-immunoprecipitation of wild type ICP27 and flag-tagged ICP27 mutants^30^. A series of ICP27 mutants with deletions and insertions in regions throughout the protein revealed that the C-terminal region of about 100 amino acids encompassing a cysteine-histidine-rich region of ICP27 was required for the self-association. Having defined the structure of the C-terminal globular region of ICP27, several residues in the binding surface were selected for mutational analysis to determine whether perturbing these residues would affect the dimerization of ICP27 during infection. To introduce unfavorable electrostatic repulsions at the interface and thus possibly disturb the dimer, residues L253, I255, D388 and T392 were individually mutated to arginine in an ICP27 plasmid with an N-terminal GFP tag. In this way, the ICP27 mutants could be distinguished from wild type ICP27 by the addition of the ~27 kDa GFP fusion protein. To confirm that ICP27 forms dimers during infection, cells were infected with wild type HSV-1 strain KOS and N-YFP-ICP27, which has an N-terminal YFP tag and which replicates as efficiently as KOS^31^. Immunoprecipitation was performed using anti-YFP antibody on nuclear lysates from cells that were infected for 8 hours. Immunoprecipitated samples were separated by SDS-PAGE and transferred to nitrocellulose. The western blot was probed with anti-ICP27 antibodies. Wild type ICP27 is not recognized by anti-YFP antibody. ICP27, which migrates at 63 kDa, and which was clearly seen in the sample from the co-infection of KOS and N-YFP-ICP27 (Fig. 6A) was co-immunoprecipitated with N-YFP-ICP27 indicating that ICP27 dimers were formed during co-infection. To determine if the C-terminal tail region of ICP27 is required for the dimerization, cells were co-infected with N-YFP-ICP27 and mutant n504. Wild type ICP27 from KOS was again co-immunoprecipitated with anti-YFP antibody however n504 was not detected (Fig. 6B), indicating that the mutant protein lacking the tail was not able to dimerize with YFP-ICP27. Next, the ICP27 point mutant plasmids described above were co-transfected with a plasmid expressing wild type ICP27. Twenty-four hours after transfection, cells were infected with ICP27 null mutant virus 27-LacZ to recapitulate the conditions of infection. At 8 hours after infection, lysates were immunoprecipitated with anti-GFP antibody and samples were analyzed by western blot analysis. All of the mutants with single amino acid substitutions along the binding surface were able to form dimers with wild type ICP27 (Fig. 6C). However, in co-transfection of an ICP27 mutant with a stop codon at amino acid 500 (STOP500) and wild type ICP27 expressing plasmid, ICP27 was not co-precipitated with the GFP-tagged mutant and thus ICP27-STOP500 was not able to form dimers with wild type ICP27. These results indicate that the C-terminal tail region (which is involved in domain swapping and dimerization in the crystal structure) is required for ICP27 to form dimers *in vivo* during infection. However, single substitutions along the binding surface did not prevent dimer formation, suggesting that the individual charge repulsions introduced by mutations were not significant enough to disrupt the dimer interaction.

## Discussion

The structure of ICP27Δ241, which encompasses a signature IHD common to all herpesviruses, revealed a novel dimeric fold comprising two α-helical bundles, each with a zinc binding site (Fig. 1). The homo-dimerization appears stabilized by a reciprocated domain-swap in which the two N-terminal helices on one chain wrap around the partner’s globular domain making extensive surface contacts, while also a short hydrophobic and aromatic-rich C-terminal tail slots into the dimeric partner and becomes covered and locked in place with a loop acting as a lid (Fig. 3). It may be anticipated that dimer dissociation and reforming (such as observed *in vivo*) may be accompanied by significant structural rearrangements in this part of the interface. The presence of the C-terminal tail region is critical for the dimer formation, as the complete or partial deletion of this region blocks dimer formation *in vivo* (Fig. 6). Interestingly, no single-point mutation of amino acid residues involved in the dimer interface, which included the introduction of repulsive charges, have any noticeable effect on the dimer formation. This can possibly be explained by the exceptionally large protein interaction interface, which was able to compensate for the introduction of unfavorable interactions at any single point. It is also possible that the major part of the binding affinity within this dimer is conferred by the C-terminal tail, which becomes largely buried upon dimer formation.

Early analyses of the ICP27 primary sequence suggested the presence of three KH domains^4^,^5^, however the experimental structure presented here clearly shows that ICP27 adopts a non KH-fold as it lacks its characteristic features such as β-sheets^32^. The zinc coordination site is also of note as it differs from early predictions. The observed CHCC site is formed by amino acid residues separated in the primary sequence, with the first coordinator C400 and the other three H479, C483 and C488 brought together in space upon protein folding. Although in principle one might anticipate that zinc may play a direct role in one of the functions of ICP27, the position of this ion at the periphery of the molecule with its charge screened from the solvent by the coordinating side chains suggests that the metal appears to have a role in stabilizing the structure of ICP27.

It has been expected that ICP27 can form a dimer, as observed here in the crystal form and in solution, as self-association of HSV-1 ICP27 have been reported previously^30^,^33^. Dimerization has also been detected for the related alpha-herpesvirus homolog VZV ORF4 protein^34^. Multimerisation in gamma-herpesviruses has also been observed in ORF57 from KSHV^35^, and recently specifically dimerization was shown to occur in its isolated C-terminal domain^36^. Additionally, EBV EB2/SM protein can self-associate^37^. Similarly, in beta-herpesviruses, self-association of UL69 from CMV has been observed with studies suggesting that the region responsible is the IHD; interestingly it was reported that purified full length UL69 can form tetramers and higher order oligomers, but not dimers^38^.

The features facilitating homo-dimerization appear to be most obviously located in the three regions which we have named arm, lid and tail (Fig. 3). The tail region of ICP27 has previously been the target of mutation studies, specifically n504 (ICP27 residues 1–504 followed by non-WT residues SSLD then a stop codon) and S18 (ICP27 residues 1-504, followed by SFIPR insert then WT C-termini -FYCNSLF); the S18 mutant had a repressive effect on self-interaction, while the n504 was not tested for this effect^30^,^39^. Interestingly both n504 and S18 mutations had a negative effect on transcription of a subset of late genes^14^,^26^,^40^,^41^. Further studies identified specific gamma-2 proteins (gC and UL47) whose expression is negatively affected by truncation of the tail region^14^,^41^. Similarly a mutation in the lid, F303N also caused a defect in virus late gene expression of specifically gC^4^. The correlation of the effects of tail and lid mutants is consistent with the ICP27 structure, due to the lid-tail proximity, in particular F303N as the side chain of F303 makes intermolecular contact with the K504 side chain burying the basic functional group from the solvent, which in turn forms an intermolecular salt bridge with E358; the burial of this ionic linkage likely strengthens the dimeric interaction (Fig. 3C(iii)). The ICP27 M15 mutant (P465L and G466E) was also defective in gene regulation functions including the activation of gamma-2 genes^42^, and this defect may be related to steric hindrance of the tail motif, as in the wild type the Pro-Gly contact F512 and form a tight turn preceding helix α9. Therefore one may assume that the mutations described above may have led to perturbing formation of the dimer, and speculate that the coordinated action of an ICP27 homodimer may be required for gamma-2 gene-specific activation functions. Mutants in the ICP27 tail also curtailed interactions with the SR-protein SRp20^13^, but did not affect binding to RNA polymerase II, so dimerization may therefore be required in some protein-protein interactions but not others^16^,^43^.

Related studies with ICP27 homologs also show a positive correlation between tail motif mutations, loss of dimerization and dysfunctions in transactivation. Mutations of VZV ORF4 amino acids in the tail sequence GKYFKC had a negative effect on transactivation, and also related experiments showed the same mutations totally abolished dimerization, especially residues equivalent to ICP27 K504, Y505, F506, Y507 and C508^34^. Similarly point mutations in the GLFF (tail) region of EBV SM/EB2 and KSHV ORF57 affected transactivation in both species and repression in the former^44^,^45^. Recently KSHV ORF57 mutants within residues equivalent to ICP27 helix α5 were shown to abolish dimerization^36^. These mutations to A or P were in residues equivalent to positions D357+E358 or W362 in ICP27^36^; from a structural perspective these changes are likely to remove some interactions with the tail region and, especially in the case of proline mutants, potentially destabilize the protein fold. Although this information is suggestive of a conserved role of dimerization in transactivation, the tail mutants may also change conformation of a binding site or destabilize the overall protein fold leading to complete loss of biological function. Carefully designed experiments are required in the future to explore the functional role of dimerization of ICP27-like herpesvirus proteins in which preserving the structure of the protein will be closely controlled.

Functionally, it can be envisioned that multiple unique binding sites positioned along the ICP27 chain, in combination with homo-dimerization, could facilitate the two protein chains to carry out different roles in close proximity and mediate multiple interactions and functions. For example, two mobile N-terminal regions of ICP27 attached to the same dimeric globular core can interact with different binding partners simultaneously. ICP27 has been shown to bind RNA through an RGG box in the N-terminal disordered region^10^ and the RGG box is also involved in the interaction of ICP27 with the splicing protein kinase SRPK1^13^,^46^, therefore a homo-dimer may be able to bind both SRPK1 and RNA, while other proteins can interact with the folded C-terminal domain. This increases the complexity of possible assemblies involving ICP27 and its homologs, and may facilitate cooperative interactions^47^. In addition, the breaking of the dimer to form monomeric species may be a possible mechanism to modulate ICP27 function *in vivo*, which still remains to be explored.

The structural data presented here for the first herpesvirus protein of its class, ICP27, showing an unusual intertwined dimeric fold, now opens the possibility for more reliable modelling for its homologues from other herpesviruses. It should also inform future functional studies by allowing the design of more targeted and rational mutants, which would not perturb the protein structure itself.

## Materials and methods

### Cloning and expression

DNA encoding an HRV3C protease cleavable N-terminal thioredoxin tag and ICP27 residues 241–512 (ICP27Δ241) was obtained by gene synthesis (Invitrogen) and codon optimized for expression in *E.coli*. Longer ICP27 constructs encoding residues 189–512 (ICP27Δ189) and 215–512 (ICP27Δ215) were also produced in the same manner. DNA was cloned into the NdeI and XhoI restriction sites of pET-21a(+) (Merek). Protein was expressed in *E.coli* strain T7 express LysY (New England Biolabs). Terrific Broth (Sigma) supplemented with 50 μg/mL ampicillin and 0.1 mM ZnSO_4_ was inoculated with 1% v/v overnight pre-culture. Culture density was monitored at 600 nm until 0.6, at which point protein expression was induced with 0.25 mM IPTG and incubation continued for 18 hours at 20 °C. Cells were pelleted by centrifugation (5000 g, 20 minutes). Selenomethionine (SeMet) labelled protein was obtained by growing cells in M9 minimal media in place of terrific broth, using the protocol described by Van Duyne *et al.*^48^.

### Protein purification

Cell pellet was resuspended in ice cold running buffer (20 mM Tris, 500 mM NaCl, 50 mM L-Arg, 50 mM L-Glu, 50 mM L-Pro, 0.1 mM TCEP, pH 8.5) supplemented with 0.5% v/v Triton X-100, DNase, RNase and EDTA free protease inhibitor (Roche). The cell suspension was lysed by sonication and clarified by centrifugation (32000 g, 30 minutes, 4 °C) then the supernatant was passed through a 0.2 μm filter. The supernatant was purified on an AKTA prime with a 5 mL StrepTrap HP column, and bound material was eluted with 5 mM *d*-desthiobiotin in running buffer. To simultaneously cleave the thioredoxin tag and remove *d*-desthiobiotin, the protein was transferred into 3.5 kDa MWCO snakeskin membrane (Pierce) with HRV3C protease (Sigma) and dialyzed for 16 hours at 4°C against 400 mL running buffer. The cleavage reaction was flowed through a clean 5 mL StrepTrap HP column in running buffer and the flow through was collected, which contained tag-free ICP27. Finally the protein was purified on a Superdex 75 26/600 column (GE healthcare) pre-equilibrated in gel filtration buffer (20 mM Tris, 150 mM NaCl, 50 mM L-Arg, 50 mM L-Glu^49^, 50 mM L-Pro, 0.1 mM TCEP, 10 μM ZnCl_2_, pH 8.5). Purified ICP27 C-terminal constructs were concentrated in Vivaspin 500 centrifugal devices with a 5 kDa MWCO (Sartorius Stedim Biotech GmbH) prior to crystallization screens.

### Crystallization

Purified selenomethionine derivatized ICP27Δ241 concentrated to 7.8 mg/mL was used to setup 5 × 96 crystal trials and screened by the sitting drop vapor diffusion method. A 200 nL drop of purified ICP27Δ241 was mixed with 200 nL of the screen condition using a TTP Mosquito Crystal nanolitre pipetting robot. Following 24 hour incubation at 4 **°**C the plates were manually inspected and a secondary round of Matrix seeded crystallogenesis was used to optimize the initial hits^50^. Seed stocks were generated by harvesting preliminary hit crystals and re-suspending them in 50 μL of mother liquor. These preliminary hit crystals were processed using a MicroSeed Bead from Molecular Dimensions (MD2-14), the sample was vortexed for 90 seconds at room temperature before being transferred to ice prior to immediate use in Matrix seeding. The Matrix seeded optimization trials involved reducing the protein volume from the initial 200 nL to 180 nL whilst supplementing the trials with 20 nL of the undiluted seed stock. Following a further 24 hour incubation at 4 **°**C single crystals suitable for X-ray diffraction analysis were observed in a range of conditions. The native protein was crystallized in a similar manner, using seed stocks generated from the initial selenomethionine derivatized crystals. Both selenomethionine derivatized and native crystals grew from reservoir solutions consisting of 0.09 M (NaF, NaBr, NaI), 0.1 M (Imidazole, MES) buffer system pH 6.5, 30% EDO_P8K mix (Morpheus HT96 B2 Molecular Dimensions) and 0.12 M (1,6-Hexanediol, 1-Butanol 1,2-Propanediol (racemic), 2-Propanol, 1,4-Butanediol, 1,3-Propanediol), 0.1 M (Imidazole, MES) buffer system pH 6.5, 30% EDO_P8K mix (Morpheus HT96 D2 Molecular Dimensions) and were flash frozen by plunge freezing in liquid nitrogen prior to data collection at Diamond Light Source Ltd.

### Data Collection, Structure Determination, Model Building and Refinement

Two data sets were collected from single cryo frozen crystals of ICP27Δ241 at i04-1 of Diamond Light Source. A single, low dose, high redundancy SeMet SAD data set was collected to 2.18 Å and a second higher dose native data set extending to 1.92 Å was collected from a second crystal (Table 1). All data were collected at a wavelength of 0.92 Å and belonged to space group P 2_1_ 2_1_ 2.

All data were scaled and processed using Xia2, and the selenium substructure and initial map calculation, as well as model building, carried out using Phenix.AutoSol^21^,^51^. The SAD phasing located six selenium sites per monomer with an overall figure of merit of 0.36 at 2.18 Å. Automated chain tracing with RESOLVE^52^ led to a model containing 513 of a possible 540 residues with a map-model correlation coefficient of 0.82 and R and R_free_ of 20.8 and 24.8% respectively (5% of reflections were selected to generate the R_free_). This initial model was subsequently rebuilt and refined using the higher resolution 1.92 Å native data with iterative cycles of rebuilding and refinement carried out in COOT and Phenix^22^,^53^. Validation with both MolProbity and PDB_REDO was integrated into the iterative rebuild process^54^,^55^. Complete data collection and refinement statistics can be found in Table 1. The deposited coordinates show a MolProbity score of 0.81 with 99% of residues falling in the favored region of the Ramachandran plot and an overall clashscore of 1.

### Biophysical characterization of ICP27 dimers

For size exclusion chromatography coupled with multi angle light scattering (SEC-MALS) analysis, samples (0.5 mL at 1 mg/mL) were loaded onto a Superdex 200 10/300GL column (GE life-sciences, 0.75 mL/min in gel filtration buffer) and passed through a Wyatt DAWN Heleos II EOS 18-angle laser photometer coupled to a Wyatt Optilab rEX refractive index detector. Data were analyzed using Astra 6 software (Wyatt Technology Corp., CA, USA). For sedimentation analytical ultracentrifugation, the protein constructs (1 mg/mL) were buffer exchanged into 20 mM HEPES pH 7.4, 150 mM NaCl by exhaustive dialysis. Sedimentation velocity was carried out using a XL-A model centrifuge at 40000 RPM at 20 °C in 450 μL double sector cells. The sedimenting boundary was monitored every 90 seconds using a wavelength of 280 nm for a total of 200 scans. Data were interpreted with the model-based distribution of Lamm equation solutions c(s) using the software Sedfit^56^. Apparent sedimentation coefficients were obtained by integration of the peak, and the hydrodynamic radius and frictional ratios (*f*/*f*_o_) for the sedimenting dimer were calculated in the program Sednterp^57^ using the mass obtained from MALS.

### Virus infection, transfection and immunoprecipitation procedures

HeLa cells were grown on minimal essential medium containing 10% newborn calf serum. HSV-1 wild type strain KOS, null mutant 27-LacZ, N-YFP-tagged ICP27 (N-YFP-ICP27) and n504 were previously described^31^. Cells were infected with wild-type or mutant virus as indicated for 8 hours at a multiplicity of infection (MOI) of 10 for single infections and a MOI of 5 for co-infections and incubated at 37 °C. Transfection of plasmid DNA was performed by using Lipofectamine 2000 reagent (Invitrogen) according to the manufacturer’s protocol. Transfected cells were infected 24 hours after transfection with 27-LacZ to stimulate expression of the native ICP27 promoter by the virion tegument protein VP16 as previously described^58^. Eight hours after infection, cells were harvested, and immunoprecipitation was performed on cell lysates using GFP/YFP antibody Ab290 (Abcam) as described previously^6^. Immunoprecipitated complexes were separated by SDS-polyacrylamide gel electrophoresis and transferred to nitrocellulose. Western blot analysis was performed as described previously^6^ with anti-ICP27 antibodies P1119 and P1113 (Virusys), anti-GFP/YFP (Ab290; Abcam), anti-GFP antibody Ab1218 (Abcam) and Lamin B1 (Abcam).

## Additional Information

**How to cite this article**: Tunnicliffe, R. B. *et al.* The structure of the folded domain from the signature multifunctional protein ICP27 from herpes simplex virus-1 reveals an intertwined dimer. *Sci. Rep.* **5**, 11234; doi: 10.1038/srep11234 (2015).

## Supplementary Material

Supplementary Information

## Figures and Tables

**Table 1:** **Data collection and refinement statistics for ICP27Δ241 structure.**

	**ICP27-Refined-Native**	**ICP27-SeMet-Solution**
**Data collection**		
Space group	P 2_1_2_1_2	P 2_1_2_1_2
Cell dimensions		
*a*, *b*, *c* (Å)	99.3, 133.6, 45.0	99.7 132.9 44.9
α, *β,* γ (°)	90, 90, 90	90, 90, 90
Resolution (Å)	46.50 - 1.92	66.46 - 2.18
	(1.99 - 1.92)*	(2.26 - 2.18)
*R*_merge_	0.061 (0.64)	0.16 (0.73)
*I*/σ*I*	19.0 (2.9)	19.0 (6.0)
Completeness (%)	100 (99)	100 (100)
Redundancy	6.6 (6.7)	26.4 (27.2)
Total reflections	305630 (30629)	842586 (85221)
Unique reflections	46524 (4553)	31964 (3138)
**Refinement**		
Resolution (Å)	46.50 - 1.92	66.46 - 2.18
Reflections used in refinement	46517 (4553)	31960 (3138)
*R*_work_	0.173 (0.248)	0.208 (0.248)
*R*_free_	0.209 (0.285)	0.247 (0.312)
*CC1/2*	0.99 (0.89)	0.99 (0.97)
No. atoms		
Protein	4165	4021
Zn^2+^ ion	2	0
Water	345	366
B-factors		
Protein	37.1	25.8
Zn^2+^ ion	25.1	N/A
Water	39.2	32.6
R.m.s deviations		
Bond lengths (Å)	0.006	0.027
Bond angles (°)	0.90	2.07
Ramachandran		
Favored (%)	99.1	99.4
Allowed (%)	0.6	0.6
Outliers (%)	0.2	0

^*^Highest resolution shell is shown in parenthesis.

**Table 2:** **Biophysical data indicates that ICP27 forms homodimers in solution in all constructs tested.**

**Construct**	**Predicted MW (kDa)**	**AUC**			**MALS**	
		***f/f***_***0***_	***S***_**20,W**_ **(S)**	**MW (kDa)**	**MW (kDa)**	***R*_h_**
ICP27Δ241	30.1	1.29	4.23 ± 0.05	58.6	60.4 ± 0.06	3.18 ± 0.02
ICP27Δ215	32.6	1.5	3.92 ± 0.06	65.7	66.7 ± 0.01	3.81 ± 0.02
ICP27Δ189	35.4	1.61	3.9 ± 0.01	72.5	73 ± 0.09	4.31 ± 0.03
