# Supplementary material for: The structure of the folded domain from the signature multifunctional protein ICP27 from herpes simplex virus-1 reveals an intertwined dimer
Source: Sci Rep. 2015 Jun 11;5:11234. doi: 10.1038/srep11234 (PMC4650695; doi:10.1038/srep11234)
Supplement: Supplementary Information [file srep11234-s1.pdf]

Supplementary information

The structure of the folded domain from the signature multifunctional protein ICP27 from herpes simplex virus-1 reveals an intertwined dimer

Richard B. Tunnicliffe[1,2], Mitchell Schacht[3], Colin Levy[1], Thomas A. Jowitt[2], Rozanne M. Sandri-Goldin[3], Alexander P. Golovanov[1,2*]

[1] Manchester Institute of Biotechnology, The University of Manchester, Manchester, UK.

[2] Faculty of Life Sciences, The University of Manchester, Manchester, UK.

[3] Department of Microbiology and Molecular Genetics, School of Medicine, University of California, Irvine, USA.

* Corresponding author. Mailing address: Manchester Institute of Biotechnology, The University of Manchester, 131 Princess Street, Manchester M1 7DN, UK. Tel. No: +44 (0) 161 306 5813; FAX: +44 (0) 161 306 5201; e-mail A.Golovanov@manchester.ac.uk

Table 1S. Intermolecular polar contacts. Inter-chain hydrogen bonds and salt bridges detected in the ICP27Δ241 structure by ePISA (Krissinel and Henrick 2007).

Hydrogen bonds

##	Structure 1	Dist. [Å]	Structure 2
1	B:ALA 420[N]	2.91	A:ASP 243[OD1]
2	B:ARG 435[NH1]	3.66	A:ASP 243[OD2]
3	B:LEU 419[N]	2.88	A:ASP 243[OD2]
4	B:LYS 423[NZ]	2.71	A:ASP 246[OD2]
5	B:ARG 480[NH2]	3.06	A:SER 272[OG]
6	B:ARG 486[NH1]	3.83	A:PRO 279[O]
7	B:HIS 502[ND1]	3.10	A:ALA 297[O]
8	B:TYR 507[N]	3.26	A:ARG 308[O]
9	B:ARG 486[NH2]	3.73	A:SER 334[OG]
10	B:ARG 486[NH1]	3.21	A:THR 335[OG1]
11	B:ARG 486[NH2]	3.27	A:THR 335[OG1]
12	B:LYS 504[NZ]	3.01	A:SER 355[OG]
13	B:LYS 504[NZ]	2.76	A:GLU 358[OE1]
14	B:TYR 505[N]	2.89	A:GLU 358[OE2]
15	B:PHE 506[N]	3.04	A:GLU 358[OE2]
16	B:THR 392[OG1]	3.55	A:ASP 388[O]
17	B:ARG 395[NE]	3.41	A:ASP 388[OD2]
18	B:ARG 345[NH1]	2.81	A:THR 392[O]
19	B:THR 392[OG1]	2.51	A:THR 392[OG1]

20	B:THR 249[OG1]	3.34	A:ILE 422[O]
21	B:LEU 511[N]	3.84	A:PRO 465[O]
22	B:SER 510[N]	2.71	A:PRO 465[O]
23	B:ARG 259[NH2]	2.94	A:GLU 474[OE1]
24	B:ARG 259[NE]	2.84	A:GLU 474[OE2]
25	B:ARG 271[NE]	2.94	A:ASP 477[OD1]
26	B:ARG 271[NH2]	3.05	A:ASP 477[OD2]
27	B:CYS 342[SG]	3.74	A:THR 491[O]
28	B:GLN 299[N]	2.94	A:VAL 501[O]
29	B:ARG 340[NH1]	2.76	A:GLY 503[O]
30	B:VAL 310[N]	2.92	A:TYR 507[O]
31	B:GLY 466[N]	3.81	A:CYS 508[O]
32	B:ARG 309[NH2]	2.97	A:ASN 509[O]
33	B:ARG 309[NH2]	2.83	A:PHE 512[O]
34	B:ASP 243[OD1]	2.91	A:ALA 420[N]
35	B:ASP 243[OD2]	2.86	A:LEU 419[N]
36	B:ASP 243[OD2]	3.59	A:ARG 435[NH1]
37	B:ASP 246[OD1]	2.65	A:LYS 423[NZ]
38	B:SER 272[OG]	3.09	A:ARG 480[NH2]
39	B:PRO 279[O]	2.97	A:ARG 486[NH2]
40	B:ALA 297[O]	3.02	A:HIS 502[ND1]
41	B:GLN 299[OE1]	2.75	A:TYR 500[OH]
42	B:ARG 308[O]	3.15	A:TYR 507[N]
43	B:SER 355[OG]	2.90	A:LYS 504[NZ]
44	B:GLU 358[OE1]	2.70	A:LYS 504[NZ]
45	B:GLU 358[OE2]	2.87	A:TYR 505[N]
46	B:GLU 358[OE2]	2.96	A:PHE 506[N]
47	B:ASP 388[O]	3.61	A:THR 392[OG1]
48	B:ASP 388[OD2]	3.46	A:ARG 395[NE]
49	B:THR 392[O]	2.88	A:ARG 345[NH1]
50	B:ILE 422[O]	3.54	A:THR 249[OG1]
51	B:HIS 461[O]	2.90	A:ARG 254[NH1]
52	B:PRO 465[O]	2.87	A:SER 510[N]
53	B:GLU 474[OE1]	2.85	A:ARG 259[NH2]
54	B:GLU 474[OE2]	2.92	A:ARG 259[NE]
55	B:ASP 477[OD1]	2.92	A:ARG 271[NE]
56	B:ASP 477[OD2]	2.98	A:ARG 271[NH2]
57	B:THR 491[O]	3.78	A:CYS 342[SG]
58	B:GLY 503[O]	2.96	A:ARG 340[NH1]
59	B:TYR 507[O]	2.88	A:VAL 310[N]
60	B:CYS 508[O]	3.69	A:GLY 466[N]
61	B:ASN 509[O]	3.02	A:ARG 309[NH2]
62	B:PHE 512[O]	2.85	A:ARG 309[NH2]

Salt bridges

##	Structure 1	Dist. [Å]	Structure 2
1	B:ARG 435[NH1]	3.66	A:ASP 243[OD2]
2	B:LYS 423[NZ]	2.71	A:ASP 246[OD2]

3	B:LYS 504[NZ]	2.76	A:GLU 358[OE1]
4	B:ARG 395[NE]	3.41	A:ASP 388[OD2]
5	B:ARG 259[NE]	3.53	A:GLU 474[OE1]
6	B:ARG 259[NH2]	2.94	A:GLU 474[OE1]
7	B:ARG 259[NE]	2.84	A:GLU 474[OE2]
8	B:ARG 259[NH2]	3.76	A:GLU 474[OE2]
9	B:ARG 271[NH2]	3.53	A:ASP 477[OD1]
10	B:ARG 271[NE]	2.94	A:ASP 477[OD1]
11	B:ARG 271[NH2]	3.05	A:ASP 477[OD2]
12	B:ARG 271[NE]	3.73	A:ASP 477[OD2]
13	B:ARG 309[NH2]	2.83	A:PHE 512[O]
14	B:ASP 243[OD2]	3.59	A:ARG 435[NH1]
15	B:ASP 246[OD1]	2.65	A:LYS 423[NZ]
16	B:GLU 358[OE1]	2.70	A:LYS 504[NZ]
17	B:ASP 388[OD2]	3.46	A:ARG 395[NE]
18	B:GLU 474[OE1]	2.85	A:ARG 259[NH2]
19	B:GLU 474[OE1]	3.36	A:ARG 259[NE]
20	B:GLU 474[OE2]	3.93	A:ARG 259[NH2]
21	B:GLU 474[OE2]	2.92	A:ARG 259[NE]
22	B:ASP 477[OD1]	2.92	A:ARG 271[NE]
23	B:ASP 477[OD1]	3.58	A:ARG 271[NH2]
24	B:ASP 477[OD2]	3.61	A:ARG 271[NE]
25	B:ASP 477[OD2]	2.98	A:ARG 271[NH2]
26	B:PHE 512[O]	2.85	A:ARG 309[NH2]

Table 2S. Intermolecular hydrophobic contacts. Hydrophobic residues in chain A that within 5 Å of hydrophobic residues of chain B are listed. Analysis performed in the ICP27Δ241 structure by PIC (Tina, Bhadra et al. 2007).

Position	Residue	Chain	Position	Residue	Chain
245	ILE	A	419	LEU	B
245	ILE	A	422	ILE	B
245	ILE	A	428	PHE	B
245	ILE	A	432	ILE	B
245	ILE	A	462	PHE	B
253	LEU	A	422	ILE	B
253	LEU	A	425	ILE	B
253	LEU	A	428	PHE	B
253	LEU	A	462	PHE	B
256	ILE	A	425	ILE	B
256	ILE	A	467	ALA	B
256	ILE	A	470	ALA	B
260	ALA	A	470	ALA	B
265	ILE	A	473	ILE	B
269	PHE	A	496	VAL	B
269	PHE	A	497	ALA	B

273	ALA	A	497	ALA	B
275	VAL	A	490	LEU	B
279	PRO	A	490	LEU	B
279	PRO	A	501	VAL	B
285	PHE	A	505	TYR	B
292	TRP	A	506	PHE	B
295	VAL	A	505	TYR	B
296	LEU	A	505	TYR	B
303	PHE	A	500	TYR	B
305	ALA	A	507	TYR	B
310	VAL	A	506	PHE	B
315	LEU	A	506	PHE	B
322	LEU	A	505	TYR	B
322	LEU	A	506	PHE	B
326	PHE	A	505	TYR	B
336	ALA	A	505	TYR	B
338	ALA	A	487	VAL	B
338	ALA	A	490	LEU	B
343	VAL	A	500	TYR	B
354	ALA	A	507	TYR	B
361	ALA	A	506	PHE	B
362	TRP	A	506	PHE	B
378	PRO	A	505	TYR	B
379	ILE	A	505	TYR	B
379	ILE	A	506	PHE	B
419	LEU	A	245	ILE	B
422	ILE	A	245	ILE	B
422	ILE	A	253	LEU	B
425	ILE	A	253	LEU	B
425	ILE	A	256	ILE	B
428	PHE	A	245	ILE	B
428	PHE	A	253	LEU	B
432	ILE	A	245	ILE	B
462	PHE	A	245	ILE	B
462	PHE	A	253	LEU	B
464	LEU	A	511	LEU	B
467	ALA	A	256	ILE	B
467	ALA	A	511	LEU	B
470	ALA	A	256	ILE	B
470	ALA	A	260	ALA	B
470	ALA	A	511	LEU	B
470	ALA	A	512	PHE	B
473	ILE	A	265	ILE	B
473	ILE	A	512	PHE	B
487	VAL	A	338	ALA	B
490	LEU	A	275	VAL	B
490	LEU	A	279	PRO	B
490	LEU	A	338	ALA	B
496	VAL	A	269	PHE	B
497	ALA	A	269	PHE	B

497	ALA	A	273	ALA	B
498	PRO	A	269	PHE	B
500	TYR	A	303	PHE	B
500	TYR	A	343	VAL	B
501	VAL	A	279	PRO	B
505	TYR	A	292	TRP	B
505	TYR	A	295	VAL	B
505	TYR	A	296	LEU	B
505	TYR	A	322	LEU	B
505	TYR	A	326	PHE	B
505	TYR	A	336	ALA	B
505	TYR	A	378	PRO	B
505	TYR	A	379	ILE	B
506	PHE	A	292	TRP	B
506	PHE	A	310	VAL	B
506	PHE	A	315	LEU	B
506	PHE	A	361	ALA	B
506	PHE	A	362	TRP	B
506	PHE	A	379	ILE	B
507	TYR	A	303	PHE	B
507	TYR	A	305	ALA	B
507	TYR	A	354	ALA	B
511	LEU	A	464	LEU	B
511	LEU	A	467	ALA	B
511	LEU	A	470	ALA	B
512	PHE	A	470	ALA	B
512	PHE	A	473	ILE	B

References:

Krissinel, E. and K. Henrick (2007). "Inference of macromolecular assemblies from crystalline state." J Mol Biol **372**(3): 774-797.
Tina, K. G., R. Bhadra and N. Srinivasan (2007). "PIC: Protein Interactions Calculator." Nucleic Acids Res **35**(Web Server issue): W473-476.
